# Comparative analysis of Australian climate change and COVID-19 vaccine audience segments shows climate skeptics can be vaccine enthusiasts

**DOI:** 10.1038/s41598-022-26959-5

**Published:** 2023-01-20

**Authors:** Lucy M. Richardson, Jagadish Thaker, David C. Holmes

**Affiliations:** 1 Monash University, Melbourne, Australia; 2 University of Auckland, Auckland, New Zealand

**Keywords:** Climate change, Infectious diseases

## Abstract

Denialism and the spreading of misinformation have occurred regarding both climate change and COVID-19, delaying uptake of urgent actions. Audience segmentation analysis identifies audience subgroups likely to have similar responses to messaging, and is a valuable tool for effective campaigns encouraging critical behaviors in both contexts. This study compared audience segmentations based on a representative sample of 1054 Australians. One segmentation was based on the ‘Global Warming’s Six Americas’ online SASSY tool. The second segmentation applied the Theory of Planned Behavior and found five distinct COVID-19 vaccine segments. Both studies showed those most concerned and those most skeptical in the climate change segmentation tended to be in more enthusiastic COVID-19 vaccine segments, while those in the center on climate change were more skeptical on COVID-19 vaccines. Differences identified relating to age, gender, and political views may be explained by a combination of the specific nature and histories of these issues. These findings have implications for effective communication on science and health issues across diverse disciplines.

## Introduction

The global nature and disastrous impacts of both COVID-19 and climate change have led several scholars to compare how these two issues have been communicated and addressed. These comparisons have examined aspects such as risk perception and associated policy responses^1,2^, social justice issues^3,4^, and the presence and impacts of misinformation and denialism^5,6^. Yet, how people perceive and respond to these two complex science and health topics may be different, even seemingly contradictory.

Evidence-informed decision-making—whether by policy-makers or private individuals—relies on more than the existence of published scientific evidence. Such decisions involve multiple knowledges, contextualization, and values-based trade-offs^7–9^. Calls must also be made on evidence quality and on what constitutes ‘enough’ evidence^7–11^. Within this complexity, the acceptance or rejection of information as evidence can vary considerably from person to person—especially with the prevalence of misinformation and denialism in a ‘post truth’ era^12^.

Misinformation and denialism have a long history in the context of communication of climate change^13^, and have also appeared in relation to the COVID-19 pandemic^14^. This misinformation and denialism have negative impacts on public responses to action on both climate change^10^ and COVID-19^14^. Research has identified effective messaging strategies for preventing or mitigating the negative impacts of misinformation^10^, but responses to these can vary across different subgroups within the population^15^. Trust in scientists and scientific consensus can also mediate susceptibility to, and impacts of, misinformation^14,16^. Trust is a multifaceted and complex concept, involving rational components as well as emotions and morals^17^. For globally significant issues such as COVID-19 and climate change, understanding the trust and distrust of science is critical for designing effective messaging to address misinformation and encourage societal action.

Distrust or skepticism of science—the systematic and unwarranted rejection of science—varies across countries and across scientific domains^18,19^. While this skepticism is relatively low in many countries, general trust in science is moderate^18^. Moreover, whilst skepticism may be practiced by a minority, this small group tends to be very vocal and influential^20^. In the case of COVID-19, science pessimism (a form of science skepticism) was associated with reduced perceptions of vaccine efficacy, increased expected vaccine risk, and reduced willingness to be vaccinated^21^. Scholars have also posited that broader audiences are becoming more critical of science, with the findings of a recent Swiss study supporting a downward trend in trust in science^22^. Conspiracy theories also run counter to accepted science, and a surprising proportion of the public across various countries accept one or more conspiracy theories as true^23^.

This study aims to explore science acceptance/skepticism by public audiences across two disparate domains—climate change and COVID-19 vaccines—as they relate to effective public campaign message targeting. According to the World Health Organization (WHO), climate change and vaccine hesitancy are two of the top 10 health threats to the world^24^. Climate change affects human infectious diseases through impacts on hosts (including human hosts and animal vectors as intermediate hosts), pathogens, and transmission^25^. While the emergence of COVID-19 has not been directly linked with climate change, its transmission to humans by infected animals^26^ is concerning. All recent pandemics have originated in wildlife due to increasing human pressure on the environment^24^, and human-wildlife transmissions are likely to increase as climate change forces increased competition for resources^25^. Vaccinations are a critical preventive tool for management of pandemic scale diseases, and were a highly topical communication focus for COVID-19 management at the time of this study. Attitudes towards COVID-19 vaccinations are likely to be different to those from vaccines such as influenza vaccines and childhood immunisations, due to its pandemic status and its newness precluding normalisation. The global urgency associated with both climate change and COVID-19 vaccinations provide an important point of intersection. Finally, as public opinion surveys attest^27^, it is possible that the public has experientially—and vicariously through media^28,29^—learned about the global scale of the problem and our collective impact on the environment when carbon emissions dipped^30^ due to national lockdowns in 2020.

Previous studies relating to public skepticism on science have largely focused on specific scientific, technology, or health domains—often those that have been controversial for some audiences. These studies have examined issues such as climate change, genetically modified foods, evolution, stem cell research, the ‘big bang’, nanotechnology, vaccines, and both COVID-19 in general or COVID-19 vaccines specifically^31–34^. While a few studies have examined correlations between skepticism on multiple topics (e.g., COVID-19 issues and climate change^34^; evolution and climate change^35^), they did not examine how various factors affected these relationships^34^. Few studies help understand what drives individuals to hold similar or contrasting views across scientific issues, or the “heterogeneity of science skepticism”^33^. In particular, this study extends previous research on social bases for environmental concern that focuses on social identities not just as statistical controls in multivariate models but as primary areas of study of social support for science^36–38^. There is increasing recognition that science and health issues are inherently social issues and scholars have called for more research on sociological determinants of public attitudes towards science^39^.

Based on a national sample of the Australian public, we identified like-minded audience segments on climate change and COVID-19 vaccine support. We examined the differences in public responses to the two issues and the effect of a range of factors on the relationship between segment membership across the two topics. We found that some individuals hold seemingly contradictory views on climate change and COVID-19 vaccines such that while they remain skeptical about climate change, they are enthusiastic about getting a COVID-19 vaccine. Age, gender, and political identity differences are reported that partly explain differences in acceptance/rejection of science across segments and domains.

### Trust, politicization, and public opinion

Public trust in science varies across topics^31,32^ and countries^18^, and is highly politicized and polarized on some issues such as climate change^40^. Actors in political debates also often politicize science through strategic use of scientific knowledge or highlighting uncertainties to promote certain policy agendas^41^. Such bias can also result from motivated reasoning, whereby expert knowledge that runs counter to an individual’s political beliefs or identity, for example, is rejected^42^, leading to selective acceptance of only that which agrees with one’s worldview and criticizing information and ideas that do not fit. Motivations behind selective reasoning are diverse, and may involve multiple goals at the same time^43^. This may explain why a range of factors such as age, gender, and political views help predict differences in individuals’ views on science, yet variably across scientific domains^31,32^.

While climate change science has long been politicized in many countries, recent studies have found similar trends with COVID-19 vaccines. The politicization of climate science in developed countries such as the US and Australia, in particular, have led to considerable public polarization^44,45^. The politicization of science surrounding COVID-19 vaccines also provides an example of how the intersection of economic and political concerns can bias the representation and acceptance of science, such as downplaying the preventative value of face masks to discourage hoarding of important protective equipment needed by healthcare workers^46^. Gender is another factor that is known to influence attitudes to science and technology. Women tend to be more skeptical of science and technology than men, and more sensitive to risk^47^. A substantial body of research has documented a small but persistent gender gap in environmental concern, including on climate change, such that women tend to be more concerned about the environment than men (see^48^ for example). Scholars have explained this gender gap through gender socialization, vulnerability hypothesis, and ethic of care, among others (see^35^ for review). Gender gaps have also been found in attitudes to COVID-19 vaccines on several continents^49–52^. Although few studies attempt to explain these differences, a US study found hesitancy due to circumspection was higher for women than men^53^, suggesting that the gendered risk-science-acceptance relationship may also apply in the context of the new COVID-19 vaccine technologies.

Another key variable is political identity. Political affiliation and ideology have been shown to be strong predictors of attitudes to climate change^40,48^ and vaccines^54^ in some countries^55^. In Australia and the US, people with conservative political affiliations and ideologies have been more likely to be skeptical of climate change^40,48^, however it is unclear how political identity relates to vaccines in Australia, or if the patterns of skepticism are similar for vaccines and climate change. This research, therefore, examines how age, gender, and political identities relate to public attitudes towards diverse scientific topics.

### Audience targeting through segmentation

Improving trust in science and countering misinformation may offer important opportunities for encouraging prosocial behaviors to address critical global issues such as COVID-19 and climate change. Research has shown that higher trust in science is associated with lower susceptibility to misinformation on COVID-19^56^ and that exposure to misinformation reduces acceptance of science on climate change^57^. Message targeting offers considerable potential to assist in addressing these issues. Targeting messages to specific audiences has been found to be an effective campaign strategy, although its application in climate change communication is considerably more recent than for health communication^58,59^. The ability to effectively target messages to specific audiences in the context of socially and scientifically complex issues such as climate change and COVID-19 vaccines—especially with regard to skepticism and misinformation—offers important value towards global survival.

Audience segmentation divides “a heterogeneous audience into relatively more homogeneous [sub-]audiences” based on common characteristics such as attitudes, needs, behaviors, etc. (p. 268)^59^. Segmentation has been undertaken for many years to assist in grouping like-minded, interpretive communities relating to climate change (see^60^ for a longitudinal example) and health (see^61^ for example). Targeting messages to such segments is a valuable tool for improving the effectiveness of communication campaigns^58,59^. However, we are not aware of any comparisons of segment membership across such diverse disciplines as health and climate change, where differences may help identify underlying factors that affect specific manifestations of faith in science and thus provide targets for improving attitudes towards science across diverse domains. For example, a US study found that lack of scientific literacy—widely believed to drive scientific skepticism—was only associated with doubt about genetic modification but not other scientific issues^33^. And a cross-country analysis found that spirituality best-predicted vaccine skepticism and low faith in science in industrialized countries^18^. That is, while it may seem intuitive that those who disregard science in one domain will disregard it in other disciplines, scholars have found that public responses vary. The factors that likely drive public skepticism in different scientific fields also vary.

However, such previous studies have used single items to measure skepticism across multiple science domains. Instead, a theory-driven segmentation approach provides a comprehensive understanding of science audiences and factors that help differentiate between them. Such research will better help clarify how different interpretive communities view multiple scientific topics and what are the social factors that help us understand how various publics make sense of—and can be better engaged with—science and health issues. An increasing number of studies in science and health communication now adopt the segmentation approach.

### Climate change and COVID-19

Climate change has evolved over decades into a highly politicized, polarizing issue in some countries^40,62^ and, although COVID-19 has a much shorter history, it too has become politically polarizing in some countries^63^. Indeed, some argue that science cannot be separated from the political, as it occurs within societal and political contexts—both informing, and informed by, politics^41,64^.

While climate change and COVID-19 are both global issues with potentially catastrophic consequences that have a substantial volume of associated scientific research and require unprecedented levels of collective action, global responses to each issue have been considerably different. In contrast to the decades of advance warning of climate change, COVID-19 appeared in the space of a few months as a more immediate and personal risk to people across the world. Climate change has been historically perceived as distant—both temporally and spatially^65^. This perceived distance, known as psychological distance, also affects COVID-19 risk perception^66^, although COVID-19 has generally been perceived as a more ‘imminent’ threat^3^. This sense of imminence led to more rapid policy responses to the virus across the world (including vaccine development) in contrast with climate change’s decades of deliberation and delayed action^3,67^. Another factor encouraging rapid responses to COVID-19 is the typically short (3 to 4 year) electoral cycles in many Western-style democracies, which demand a resolution of the perceived immediacy of COVID-19 but not of climate change. Indeed, the rapid development of COVID-19 vaccines likely caused increased perceived vaccine risk and hesitancy towards being vaccinated for some of the public^21^. There has been considerable media spotlight on medical science throughout the pandemic, however the evolving understanding of the virus and its management, and conflicting expert recommendations, have resulted in ambiguity such as has been seen regarding climate change policy—especially when paired with the value-laden trade-offs inherent in policy decision-making^3^.

Our primary research focus was to understand the similarities and differences in how people make sense of and respond to complex scientific and health issues. We chose to focus specifically on COVID-19 vaccines and climate change as both were highly topical at the time of the study, with COVID-19 vaccine roll-outs beginning to occur across the globe and the United Nations Framework Convention on Climate Change’s 26th Conference of the Parties approaching at the time of surveying.

### Segmentation for climate change communication

Many climate change segmentation studies have been done over the years (see^68^ for an examination of these). The most comprehensive temporal tracking of climate change segments is *Global Warming’s Six Americas* undertaken by researchers at Yale and George Mason Universities. Their and related methodologies have been applied across a range of countries such as Australia, Singapore, and India^68^. Yale University has also published an online tool that automates segmentation using the 4-item SASSY questionnaire^69^.

Segmentation of Australian audiences by Morrison, Parton, and Hine^70^ using latent class analysis on questions from Global Warming’s Six Americas identified six Australias that align with the six segments found in the American studies: Alarmed, Concerned, Cautious, Disengaged, Doubtful, and Dismissive. The issue engagement of these segments follows a curved relationship whereby segments towards either extreme are more engaged (with opposing acceptance/rejection of the science) and those towards the center are least engaged and most uncertain about the science^70^. For the purposes of this study, we will reference climate change segments closer to Alarmed as more accepting/less skeptical of science, and those closer to Dismissive as more skeptical/less accepting.

### Segmentation for health communication

While audience segmentation has been applied in health communication for decades (see^46^ for example), few segmentation studies have been conducted relating to COVID-19 behaviors. Previous studies have examined audience segments based on conspiracy beliefs about the COVID-19 disease origin^71^ as well as COVID-19 protective behaviors in the US^72^ and Australia^73^. Few studies have examined audience segmentation incorporating intentions to get a COVID-19 vaccine and have used a theoretical, behavioral framework to understand distinct audience segments in response to COVID-19. Studies conducted in Taiwan^74^ and New Zealand^75^ are two that did both.

These studies adopted different theoretical bases and found different numbers of segments in each nation. Three segments were identified in Taiwan based on Protection Motivation Theory, addressing a range of COVID-19 protective behaviors including vaccination: high motivation for vaccination and preventive behaviors; low motivation for vaccination and preventive tasks; and high motivation for vaccination but low motivation for preventive behaviors^74^. In New Zealand, four segments were found based on the Theory of Planned Behavior, addressing COVID-19 vaccination intentions: Enthusiasts (36%), Supporters (28%), Hesitants (24%), and Skeptics (12%)^75^.

This study examined relationships between segment memberships with regard to engagement-skepticism associated with climate change and COVID-19 vaccines using an Australian sample to better understand how consistent are the patterns of skepticism/acceptance of science across these two disparate domains. Our study aimed to answer two key research questions:

- How similar are individuals’ segment memberships with regard to acceptance/skepticism of science across audience segmentations based on attitudes to COVID-19 vaccines and climate change?
- How do individuals’ age, gender, and political identities relate to their segments across these two issues?

## Results

### Climate change segments

Twenty-six percent of the sample were in the *Alarmed* climate change segment, 31% were *Concerned*, 28% were *Cautious*, 2% were *Disengaged*, 4% were *Doubtful*, and 8% were *Dismissive*. Table 1 shows each segment’s mean response to the four relevant survey questions used for climate change segmentation. This segmentation suggests an upward shift towards higher public concern about climate change when compared with the analyses by Morrison, Parton and Hine^70^ analyzing 2011 and 2016 data based on the Global Warming’s Six Americas 36-item survey. For example, both earlier surveys showed lower membership of the Alarmed segment (18% in 2011 and 15% in 2016) and higher membership of the Dismissive segment (6% in 2011 and 11% in 2016).

**Table 1:** Descriptive statistics of climate change beliefs and concern across the segments.

	Mean	SD	Alarmed	Concerned	Cautious	Disengaged	Doubtful	Dismissive
How worried are you about climate change? (4-point Likert scale; Not at all to Extremely)	2.87	0.878	3.83	3.10	2.67	2.32	1.67	1.25
How much do you think climate change will harm you personally? (5-point scale; Not at all to Very much, with Don’t know = 0)	2.55	0.988	3.50	2.74	2.28	2.40	1.42	1.09
How much do you think climate change will harm future generations of people? (5-point scale; Not at all to Very much, with Don’t know = 0)	3.26	0.932	3.97	3.86	2.87	0.00	2.08	1.00
How important is the issue of climate change to you personally? (5-point Likert scale; Not at all to Extremely)	3.06	1.099	4.43	3.20	2.66	2.93	1.69	1.32

*N* = 1051. Table presents mean values of key variables across the segments.

The six climate change segments differed across a range of demographic characteristics (see Supplementary Table S1 for detailed distributions). The Alarmed segment tended to have relatively even distributions of gender and age. The Concerned and Cautious segments included more women than men and were generally younger, while the Doubtful and Dismissive segments were predominantly men and older. The Disengaged segment tended to be largely women, and aged in their 40s and 50s.

Political differences also existed. The majority of the sample categorized themselves as politically moderate, however there was a small to moderate correlation between segment membership and political identity (*r* = 0.168, *p* < 0.001), with those in the more skeptical segments tending to be more conservative and those in the less skeptical segments tending to be more liberal.

### COVID-19 vaccine segments

Vaccine *Enthusiasts* (28%) show most favorable attitudes towards a COVID-19 vaccine, perceive strong social norms about vaccination, and almost all say they will ‘definitely’ get a vaccine to protect themselves. Vaccine *Supporters* (26%) also have favorable COVID-19 vaccine attitudes and strong social norms towards vaccination, but significantly less than the Enthusiasts. Vaccine *Socials* (20%) have lower favorable attitudes, norms, and intentions to get a COVID-19 vaccine, but are willing to get a vaccine to protect others. Vaccine *Hesitant* (15%) have low favorable attitudes towards COVID-19 vaccines, perceive weaker social norms for vaccination, and a majority in this segment say they are “unsure, but leaning towards No,” regarding getting a vaccine to protect themselves and to protect others. Vaccine *Skeptics* (10%) have the least favorable attitudes towards a COVID-19 vaccine, perceive fewer social pressures towards getting vaccinated, and are overwhelmingly likely to ‘definitely not’ take a vaccine to protect themselves or to protect others (see Tables 2, 3). The details of the vaccine segments have been previously reported elsewhere (Thaker et al., 2022^76^ )﻿. For the purposes of this study, we will reference vaccine segments closer to Enthusiasts as more accepting/less skeptical of science, and those closer to Skeptics as more skeptical/less accepting.

**Table 2:** Descriptive statistics of COVID-19 vaccine attitudes, social norms, and behavioural control across the segments.

	Mean	Std. deviation	Enthusiasts	Supporters	Socials	Hesitant	Sceptics	*R*^2^
**COVID-19 vaccine attitudes**								
To what extent do you feel that getting a COVID-19 vaccine will be… (7-point bipolar scale)								
Bad: good	4.96	2.12	6.94	5.75	4.18	3.46	1.22	0.70
Unpleasant: pleasant	4.07	1.90	5.55	4.01	3.85	3.44	1.49	0.38
Harmful: beneficial	4.85	2.12	6.95	5.32	4.22	3.45	1.18	0.68
Worthless: valuable	4.97	2.08	6.94	5.55	4.24	3.63	1.54	0.64
Ineffective: effective	4.99	2.04	6.84	5.53	4.42	3.68	1.53	0.62
Unsafe: safe	4.59	2.06	6.64	5.01	3.94	3.28	1.09	0.66
Undesirable: desirable	4.67	2.12	6.84	4.98	4.11	3.18	1.20	0.66
**Social norms**								
How much do you agree or disagree with the following statements? (5-point scale, strongly disagree to strongly agree with neither as mid-scale)								
Descriptive norm	3.82	1.27	4.81	4.41	3.53	2.52	2.00	0.60
Subjective norm	3.91	1.20	4.87	4.57	3.54	2.68	2.11	0.67
Injunctive norm	4.06	1.15	4.89	4.59	3.60	2.90	3.05	0.47
**Behavioural efficacy and control**								
Self-efficacy	3.68	1.46	4.92	4.57	3.21	1.99	1.34	0.78
Behavioural control	4.27	1.04	4.66	4.46	3.76	3.81	4.39	0.13

*N* = 1054. Table, sourced from Thaker et al., 2022^76^ presents mean values of key input variables across the segments.

All mean differences—as judged by ANOVA or chi-square tests—are significant at *p* < 0. 001. *R*^2^ represents how much of the variance of each indicator is explained by this 5-cluster model.

**Table 3:** Question responses on COVID-19 vaccine intentions across the segments.

	Total responses (%)	Enthusiasts (%)	Supporters (%)	Socials (%)	Hesitant (%)	Sceptics (%)	*R*^2^
**Would you accept the vaccine for yourself?**							0.62
No, definitely not	14	< 0.1	< 0.1	1.3	35.1	83.7	
Unsure, but leaning towards NO	14	< 0.1	0.2	15.3	64.6	14.9	
Unsure, but leaning towards YES	17	< 0.1	9.4	70.7	< 0.1	1.3	
Yes, definitely	55	99.9	90.4	12.7	0.3	0.1	
**Would you accept the vaccine if it meant protecting friends, family, or at-risk groups?**							0.48
No, definitely not	8	< 0.1	< 0.1	0.5	16.7	53.4	
Unsure, but leaning towards NO	15	0.6	< 0.1	16.1	51.2	37.2	
Unsure, but leaning towards YES	17	0.2	4.2	52.7	26.5	7.7	
Yes, definitely	60	99.2	95.8	30.8	5.7	1.7	
**Would you be willing to put your name on the list to be vaccinated first?**							0.47
No, definitely not	20	0.4	1.5	9.9	59.0	89.3	
Unsure, but leaning towards NO	14	0.3	7.4	23.5	37.9	10.3	
Unsure, but leaning towards YES	19	5.2	18.3	59.1	3.0	0.4	
Yes, definitely	47	94.1	72.9	7.5	0.1	< 0.1	
**I will get vaccinated against the coronavirus**							0.80
No	30	1.6	< 0.1	22.41	98.9	99.9	
Yes	70	98.4	99.9	77.6	1.2	0.1	

*N* = 1054. Table, sourced from Thaker et al., 2022^76^ presents percentages of key input variables across the segments.

All proportional differences—as judged by ANOVA or chi-square tests—are significant at *p* < 0.001. *R*^2^ represents how much of the variance of each indicator is explained by this 5-cluster model.

The five COVID-19 vaccine segments differed on gender and age (see Supplementary Table S2 for detailed distributions). Vaccine Enthusiasts are overwhelmingly men (71%), whereas vaccine Hesitants were predominantly women (75%). Older respondents were most likely to be vaccine Enthusiasts (over three quarters of the segment aged 50 years and older).

These segments also showed slight to moderate correlations with political identity (*r* = 0.154, *p* < 0.001). While the majority of the sample identify as politically moderate (from 36% for Enthusiasts to 65% for the Hesitant), liberal respondents were slightly more likely to be in the more accepting segments, and conservatives in the more skeptical segments.

### Segment membership comparison

Participants’ membership in the two different segmentations tends to follow a v-like relationship based on maximum (mode) membership, where the most and least skeptical climate change segments (Alarmed and Dismissive) tended to more likely be vaccine Enthusiasts, and those in the center on climate change were likely to have a less accepting vaccine segment such as Social or Hesitant (see Table 4). The Alarmed climate change segment’s largest COVID-19 vaccine segment is the Enthusiasts (38%), however the Doubtful and Dismissive participants are also more likely to be vaccine Enthusiasts than any other vaccine segment (33% and 35%, respectively). The Concerned segment’s largest vaccine segment are Supporters (29%), and for the Cautious it is the vaccine Socials (28%). The Disengaged climate change segment has most members in the vaccine Hesitant segment (48%). There was a small predictive relationship between climate change and vaccine segments, *V* = 0.146, *p* < 0.001.

**Table 4:** Number of respondents within each combination of COVID-19 vaccine and climate change segments.

	Climate change segment					
COVID-19 segment	Alarmed (n = 240)	Concerned (n = 319)	Cautious (n = 303)	Disengaged (n = 21)	Doubtful (n = 103)	Dismissive (n = 62)
Enthusiast (n = 294)	**92**	84	58	4	**34**	**22**
Supporter (n = 277)	72	**92**	79	2	14	18
Social (n = 214)	41	53	**85**	2	27	6
Hesitant (n = 154)	18	55	53	**10**	14	4
Skeptic (n = 109)	17	35	28	3	14	12

Bold values contain the largest vaccine segment proportion within each climate change segment.

Additional analysis of the relationships between segments for men and for women showed slightly stronger predictive relationships, *V*_men_ = 0.168, *p* < 0.001, *V*_women_ = 0.192, *p* < 0.001. Comparison of cross-segment membership for men and women (see Table 5) shows substantial variability in membership patterns between these two gender identities, χ^2^ (20) = 8697.434, *p* < 0.001. In contrast to the aggregate results, men’s maximum (mode) vaccine membership across all climate segments (except Cautious) is the Enthusiast segment (39% to 50% of climate segment), except for the climate Cautious (mode was 32% as vaccine Supporters). In contrast, while sample sizes are low for women in the more skeptical climate segments, women’s maximum (mode) vaccine membership follows a V shape such that women Alarmed on climate change are most likely to be vaccine Enthusiasts (30%), women in the Concerned and Dismissive climate segments are most likely to be vaccine Supporters (28% and 60%, respectively), climate Cautious and Doubtful women are most likely to be vaccine Socials (32% for both), and climate Disengaged women are most likely to be vaccine Hesitant (69%). This shows dramatic differences in attitudes to climate change and the vaccines between genders, with men much more consistently accepting of COVID-19 vaccines than their broadly spread attitudes to climate change, while women at the extreme (accepting and skeptical) on climate change are accepting of the vaccines, and women in the center on climate change are more skeptical of the vaccines.

**Table 5:** Number of men and women respondents within each combination of COVID-19 vaccine and climate change segments.

Climate change segment	Gender	COVID-19 segment				
		Enthusiast	Supporter	Social	Hesitant	Skeptic
Alarmed	Men	**58**	45	15	8	5
	Women	**33**	27	26	10	12
Concerned	Men	**59**	34	16	5	6
	Women	24	**53**	37	50	29
Cautious	Men	37	**43**	28	18	6
	Women	21	36	**54**	35	22
Disengaged	Men	**4**	2	0	1	1
	Women	0	0	2	**9**	2
Doubtful	Men	**30**	12	14	5	3
	Women	4	3	**13**	9	12
Dismissive	Men	**20**	12	4	3	12
	Women	2	**7**	1	1	0

Bold values contain the largest vaccine segment membership (the mode value) within each climate change segment.

Review of segment relationships across different age subgroups also showed nuances that were masked in the aggregate cross-segment analysis. Each age group showed small to moderate relationships between their vaccine and climate change segments (*V*_18–29 years_ = 0.221, *p* = 0.001, *V*_30–49 years_ = 0.211, *p* < 0.001, *V*_50+ years_ = 0.190, p < 0.001). Comparisons of cross-segment distributions between age groups showed significant differences between 18 to 29 year-olds and both 30 to 49 year-olds (χ^2^ (20) = 1718.673, *p* < 0.001) and those of 50 or more years (χ^2^ (20) = 9546.343, *p* < 0.001) and between 30 to 49 year-olds and those over 50 (χ^2^ (20) = 6245.849, *p* < 0.001). Older people tended to have their maximum (mode) vaccine membership in the vaccine Enthusiast segment across the Alarmed (53% of climate segment), Concerned (57%), and Cautious (40%) climate segments. The dominant vaccine segment for younger participants tended to be more skeptical than their elders, with vaccine Supporters highest for the climate Alarmed (32%), vaccine Hesitants for the climate Concerned (32%), and vaccine Socials for the climate Cautious (40%). Participants in the middle age group tended to either match that of younger participants (for Alarmed and Cautious climate segments) or sit between younger and older participants (for the climate Concerned). Thus, older people were more consistent in their accepting attitudes towards the vaccine than their climate change attitudes, younger people’s attitudes on each issue tended to be less consistent and was more vaccine skeptical, and the attitudes of those in the middle age group tended to be somewhat more consistent across issues and between the other groups with respect to vaccine acceptance. Sample sizes in the more skeptical climate segments were very small for the two younger age groups and so are not discussed here.

Participants of different political identities did not show consistent relationships between cross-segment memberships. Conservative participants showed no significant relationship between climate change and vaccine segments, *V* = 0.136, *p* = 0.260. In contrast, politically moderate and liberal participants showed small to moderate relationships between segments, *V*_moderate_ = 0.186, p < 0.001, *V*_liberal_ = 0.216, *p* = 0.003. Comparison of segment memberships between conservative, moderate, and liberal political identities showed significant differences in the pattern of membership across all combinations: between conservatives and moderates (χ^2^ (20) = 940.414, *p* < 0.001), conservatives and liberals (χ^2^ (20) = 5157.123, *p* < 0.001), and between moderates and liberals (χ^2^ (20) = 3603.422, *p* < 0.001). The climate change segments with the highest (mode) vaccine segment membership for those who were politically liberal and moderate tended to be Enthusiasts (41% to 78% for liberals, and 29% to 39% for conservatives), except for the climate Cautious, where liberals tended to be vaccine Supporters (50%) and conservatives tended to be vaccine Socials (29%). In contrast, politically moderate participants were most likely (mode) to be vaccine Supporters (29% to 39%), except for the climate Cautious who were more likely to be vaccine Socials (31%), and the climate Doubtful who were more likely to be vaccine Enthusiasts (31%). Thus, except for the climate Cautious, liberals and conservatives had much the same modal memberships and were both more accepting of the vaccines than were the politically moderate. We do not report the Disengaged climate segment here due to its small sample sizes in some categories.

An examination of the interaction effects of gender and political identities showed further nuances. Significant relationships were found between the climate change and vaccine segments of politically moderate men (*V* = 0.288, *p* < 0.001), moderate women (*V* = 0.240, *p* < 0.001), and liberal women (*V* = 0.314, *p* = 0.005), but not for conservative women (*V* = 0.222, *p* = 0.052). The sample sizes were too small to confirm the significance of this relationship for conservative men and liberal men (post hoc power less than 80%). Due to the complexity of these interactions and the number of possible interactions to report, the results for the Concerned climate change segment are reported here as an example. The Concerned climate change segment is the largest of the climate change segments and exhibits some of the more substantial interaction effects.

**Figure 1:**
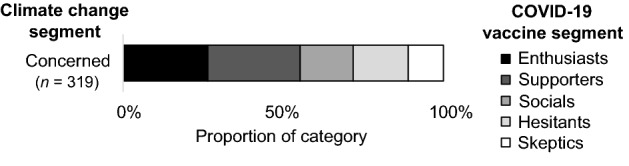
Vaccine segment distributions within the Concerned climate segment.

**Figure 2:**
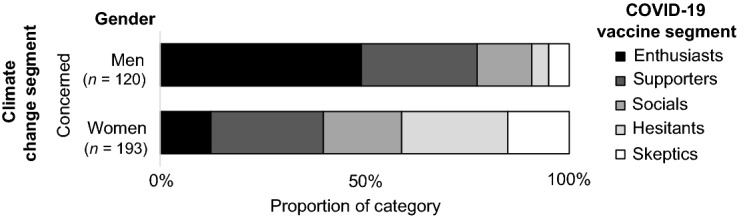
Vaccine segment distributions within the Concerned climate segment for men and women.

**Figure 3:**
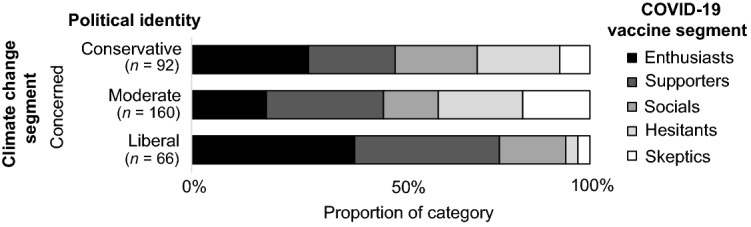
Vaccine segment distributions within the Concerned climate segment for participants with conservative, moderate, and liberal political identities.

**Figure 4:**
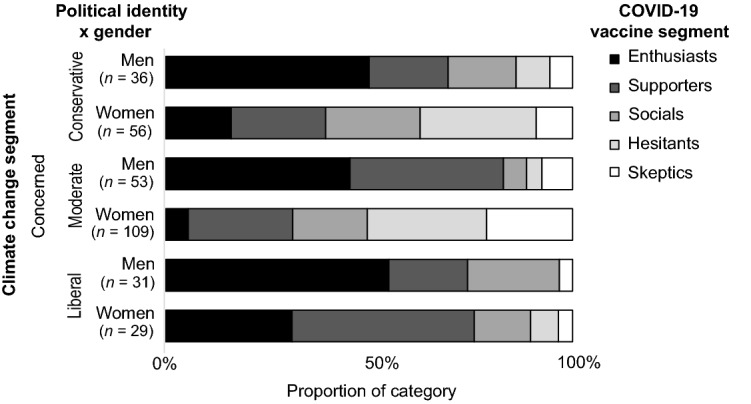
Vaccine segment distributions within the Concerned climate segment for men and women with conservative, moderate, and liberal political identities.

Figures 1, 2, 3 and 4 shows the distribution of vaccine segments within the Concerned climate change segment from the aggregate level, to single subcategories (e.g., political identity), to interactions between subcategories (e.g., gender and political identity). Comparing the aggregate result (Fig. 1) with the gender (Fig. 2) and political identity (Fig. 3) breakdowns for the Concerned climate segment illustrates the earlier discussed differences in vaccine hesitancy. The distribution across interacting gender and political identity categories (Fig. 4) shows that men appear to have somewhat similar patterns of cross-segment membership across political identities, although small sample sizes mean we can’t be confident in this result (post hoc power less than 80%). Women, however, have much more variability across political identities and sufficient sample size for confidence in these differences. Politically moderate women in the Concerned climate segment have a considerably higher proportion of members in vaccine Hesitant (29%) and Skeptic (21%) segments and the lowest proportion of vaccine Enthusiasts (6%), followed by conservative women (16%), with liberal women having the highest proportion of both vaccine Enthusiasts (31%) and Supporters (45%) and the lowest proportion of vaccine Hesitants (7%) and Skeptics (3%).

**Figure 5:**
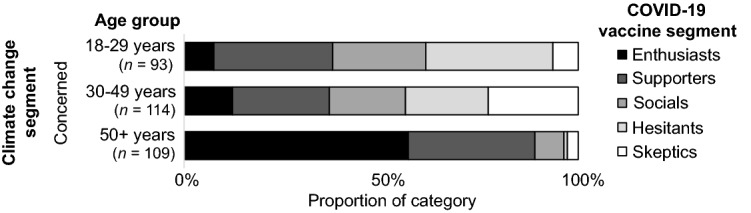
Vaccine segment distributions within the Concerned climate segment for each age group.

**Figure 6:**
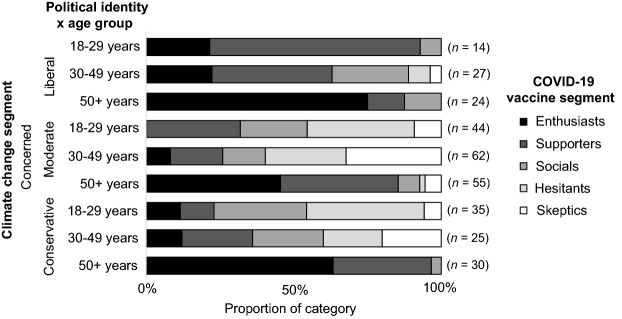
Vaccine segment distributions within the Concerned climate segment for politically conservative, moderate, and liberal across each age group.

Similar analysis of interactions between age and political identity within the Concerned segment found significant differences in vaccine segments across age groups for people with each political identity (*χ*^2^_conservative_ (8) = 48.262, χ^2^_moderate_ (8) = 72.653, χ^2^_liberal_ (8) = 25.340, all *p* ≤ 0.001). For those aged 50 years or more, however, the sample size was too small to be confident in differences across political identities (post hoc power less than 80%). Figure 5 shows the distribution of vaccine segments for those in the Concerned climate change segment for each age group, and Fig. 6 shows the interaction of these with political identity. Within the Concerned climate segment’s youngest age group, liberals tended to have the highest proportion of vaccine Enthusiasts (21%) and Supporters (71%) compared with moderates and conservatives. Similarly, the largest proportion of Enthusiasts (21%) and Supporters (41%) in the 30–49 years age group were politically liberal, and the largest proportion of Skeptics were politically moderate (9%).

## Discussion

The aim of this study was to understand how people make sense of climate change and COVID-19 vaccines—two critical science and health issues—using an audience segmentation approach. Segmentation based on participants’ climate change beliefs identified six segments from the most worried (Alarmed) segment to the most skeptical (Dismissive) segment. The majority of people were on the concerned end of the spectrum that included the Alarmed, Concerned, and Cautious segments (85%), while only 8% were actively Dismissive of climate change. As found in a range of other studies, young people and women tended to be more concerned about climate change. This is common in English-speaking, Western democracies, but is not the case in all countries^77^.

While the climate change segmentation questions used in this study have been used effectively for Australian and other audiences in the past^68,70^, it may be time to develop new approaches. The low proportion of Disengaged, Doubtful, and Dismissive climate change segments supports the findings of other studies that show the Australian public now generally accepts that climate change is real and that it is a cause for worry^78–80^. This suggests a need to shift climate change messaging away from convincing the public that climate change is real and of concern, towards helping communities make informed personal and policy decisions for addressing climate change. The four questions used to segment the public in this and other studies^80,81^ were drawn from larger survey instruments containing 15 and 36 items^82^. While some of the additional questions in these broader surveys may offer important segmentation insights in current societal contexts (e.g., questions on desired level of government priority given to climate change), others may be less relevant (e.g., acceptance that climate change is happening). Further research should examine which questions may better identify interpretive communities (segments) suitable for developing behavior- and policy-relevant messaging strategies to reflect this shift in communication priorities.

The COVID-19 vaccine analysis identified five segments on a continuum from those most supportive of these vaccines (Enthusiasts) to those most resistant (Skeptics). The sample was relatively evenly distributed across the segments, however the three more accepting segments (Enthusiasts, Supporters, and Socials) made up the majority of the sample (74%), with only 10% in the more actively Skeptic segment. Since the survey was conducted, 90% of Australians over the age of 5 have received at least one dose of a COVID-19 vaccine^83^. As this includes 96% of people aged 16 or over, it is likely that even most of the Hesitant segment have now accepted a vaccine—either as a result of change of mind or because of government mandates.

Comparison of the relationship between participants’ climate change and COVID-19 vaccine segments confirmed differences across the domains such that positive beliefs and attitudes on one issue do not equate to positive beliefs and attitudes on the other. Examining the pattern of membership across segments showed that there were predictive relationships between climate change and vaccine segment membership, and that these varied with age, gender, and political identity. This is an important finding, as it highlights some critical factors that influence issue-specific attitudes and acceptance of science.

The identified cross-issue differences in science skepticism are likely related to underlying attitude ‘roots’, which are “the factors that drive and sustain surface skepticism about science, and include deeply held worldviews, identities, and ideologies” (p. 277)^84^. Research on science skepticism is trying to better understand these attitude roots and their influence across scientific domains. While this study did not examine generations specifically (but age groups), the concept of generational identities is not new and these are known to intersect with other forms of identity^85^. Differences associated with generational identities may contribute as attitude roots reflected in the age group effects we identified. Self-identified generational identity differences in attitudes to issues like climate change have been evident in public discourse features such as the ‘Okay Boomer’ meme phenomena that began in 2019, which was used by Millennials and the iGeneration to show their frustration with the Baby Boomer generation—including the Boomers’ lack of climate action^86^. Young adults’ neuro-developmental tendency towards risk-taking^87^, combined with early perceptions that younger people were immune to COVID-19, likely formed key attitude roots explaining the more skeptical vaccine segments of young people, which contrasted with their higher acceptance of climate change science.

The socialized values and expectations associated with gender identities may also play a role in explaining the gender effects of this study. Women tend to be more skeptical of science and technology than men, and this is believed to be associated with women’s generally higher risk aversion^87,88^ and the differences in interpretive context brought about by gender-specific life views that are formed during socialization^88^. Risk perceptions are also known to vary across domains with, for example, health and safety risk perceptions typically higher than financial risk perceptions^88^. Similar to previous studies^51,89^, we found women to be more skeptical of COVID-19 vaccine technology, as evidenced by higher vaccine skepticism than men. In contrast, men tended to be more skeptical on climate change. This supports the idea that gendered risk attitudes may be an important driver of science acceptance/skepticism. The seemingly contradictory results across climate change and COVID-19 vaccines may appear counter-intuitive, however risk aversion in the case of climate change may stem from assessments that the risks posed by climate change itself overwhelm any risks associated with specific technologies that might be used to combat climate change. For women, who are often socialized towards caregiving roles, it makes sense that they would be more concerned about a societal issue such as climate change than men, although research has shown that these gender differences are more likely to be found in affluent societies^90^.

The scale and scope of each issue under consideration also provides important context for attitudes towards risks and thus acceptance of science, as risks associated with more general targets (community risk) are evaluated somewhat differently than when the self is the risk target (personal risk)^91^. Evaluations of both COVID-19 vaccines and climate change could be assessed as either a personal or general risk depending on the individual’s context and worldviews. Climate change would likely be assessed as a general risk by most people who had not yet been directly affected, but could be considered a personal risk if climate change impacts are directly posed (e.g., in a bushfire prone area) or if climate change mitigation measures are seen as impacting on one’s identity (e.g., through altering one’s consumption lifestyle). Similarly, COVID-19 vaccines could be assessed as a personal risk to one’s health (positively or negatively) or a community risk (positive or negative depending on one's conspiracy beliefs). Understanding how these risk perceptions relate to age, gender, and political identities, and how the framing and presentation of science might influence both these risk perceptions and acceptance of the science, would provide important guidance for science communication and messaging regarding action on key societal issues.

Political identities and their associated worldviews have been examined as drivers of climate change science acceptance/skepticism through the mechanism of motivated reasoning^35^. There are also cultural meanings associated with acceptance/rejection of science that impact on the social relationships attached to our identities^35^. By considering science in light of expectations and values associated with one’s political identity, one tends to rationalize the acceptance or rejection of the science in a way that supports these values and expectations, maintains one’s sense of identity, and poses no threat to relationships based on that identity. This is particularly important for politically controversial issues such as climate change and now COVID-19 and COVID-19 vaccines, where going against the expectations of important others can threaten valued relationships and inclusion in valued groups.

Worldviews evolve over time as the issues facing the world (and the individual) evolve, and individuals hold multiple, potentially conflicting, worldviews of varying strength, which are drawn upon in different contexts^92^. Better understanding how the (potentially very different) worldviews associated with age, gender, and political identities intersect, and how they manifest as attitude roots in the context of acceptance/rejection of science across different issues, would offer guidance for framing effective messaging that integrates and addresses the critical needs of each worldview in a way that supports effective decision-making in the face of the world’s wicked problems.

The complex interplay of age, gender, and political identities across the two studied domains offer important insights in support of attitude roots influencing attitudes towards climate change and COVID-19 vaccines and the acceptance of associated science. Understanding how these factors manifest across domains can help develop theories that can guide strategies for messages targeting audience segments on critical societal issues. Knowing which key attitude roots might influence science attitudes on a specific issue, and how, would allow more targeted audience research and, in turn, more effective communication.

Recent years have seen fundamental shifts towards increasingly critical community views of science^22^. Political partisanship, cultural group thinking, and motivated reasoning, all play important roles in the increasing hold of ‘post-truth’ politics and the diminishing reliance on science for collective decision-making^93^. Our research shows that views of science vary across issue areas, and that factors such as age, gender, and political identity interact in complex ways that explain some of the differences across issue areas. This has critical implications for communicators and decision makers operating within these issue areas and highlights the importance of understanding likeminded (and other-minded) audiences when designing messages.

### Limitations

Two key limitations of this study are its cross-sectional nature and the limited sample sizes for certain segments and subsegments. Longitudinal and experimental tests can help identify how audience segments align and differ on different scientific issues. Apart from the focus of this study on a few social and political identities, future research can test a more comprehensive model including conspiratorial mindset, trust, scientific knowledge, and general public attitudes towards science and technology. Our findings confirm that some individuals may hold very different views across scientific issues. For societal issues where misinformation abounds and public action is critical, future research should continue to investigate how segmentation can be used to support effective communication to address misinformation and encourage action. Future research should also continue to explore the underlying factors that lead to susceptibility to misinformation or acceptance of science across multiple domains, to consider the potential for cross-domain impacts on science acceptance.

The issue of small sample sizes includes fringe views and associated potential psychographic skew. It is possible that some combinations of identities and some segments were either overlooked within the quota sampling process, or are fringe views. These smaller subpopulations may have not been thoroughly captured and represented through the demographically-based quotas sampled, however both COVID-19 and climate change could be addressed through majority action, so small, fringe populations become less critical as long as they do not deter the majority. Future studies could incorporate nested quotas or proportional probability sampling to specifically assess the prevalence of these identities/views.

## Conclusion

This study examined the similarities and differences in how people make sense of and respond to complex scientific and health issues such as climate change and COVID-19. We confirmed that acceptance of science in one domain does not necessarily translate to acceptance in another domain, and that factors such as age, gender, and political identity are associated with differences in these levels of acceptance. This has critical implications for both theory and practice. From a theoretical perspective, our findings offer some support for the idea that various identities and their associated worldviews may form attitude roots that underpin acceptance of science, potentially manifesting through motivated reasoning and risk assessments. These mechanisms may explain why misinformation can be believed above science in certain contexts, and offer an important line of investigation for further research. Integrating such underlying beliefs and biases into audience segmentation, message targeting, and addressing misinformation, could improve the uptake of key interventions targeting complex scientific and health issues such as climate change and COVID-19.

## Participants and methods

A survey of the Australian public (*n* = 1054) was conducted between 20 May and 12 July 2021. This project was deemed low risk to participants by Massey University’s Human Ethics Committees (Project ID 4000024273) and registered with the Monash University Human Research Ethics Committee (Project ID 28612). It was conducted in accordance with relevant ethics guidelines and regulations, and all participants were fully informed and consented to their participation.

The survey was fielded by Qualtrics, which maintains an active online panel that is representative of the Australian population^94^. Participants receive an incentive such as a flat fee or discount gift card based on the length of the survey, their specific profile, and target acquisition difficulty. Respondents for this survey were sent a secured, individualized email link. The average time to complete the survey was 25 min.

The sample was slightly younger, with slightly more women, and individuals belonging to higher education and income groups when compared with national population estimates. The geographic distribution of respondents generally matched national estimates, however, there were fewer respondents from New South Wales (27% compared to national estimate of 32%). Post-survey weights were applied to align the sample with national estimates of gender and age^95^, education^96^, income^97^, and geographic distribution^98^. Hot-deck imputation^99^ was used to impute the few missing values based on gender and age parameters. No psychographic variables were included in the weightings.

Climate change segmentation was conducted on responses to the Global Warming’s Six Americas Short Survey 4-item instrument^81^ analyzed using the online SASSY! segmentation tool^69^ as has been previously applied in Australia^80^, yielding six segments. COVID-19 vaccine segmentation was conducted using Latent GOLD® software (version 5.1) on a suite of 16 questions based on the Theory of Planned Behavior (see^100^ for example), examining attitudes towards vaccines, social norms, perceived behavioral control, and intentions to receive a vaccine. Comparison of model fit, parsimony and segment sizes of models with two to eight segments identified the best model as containing five COVID-19 vaccine segments. See Supplementary Material for a full list of the segmentation questions.

Comparisons of distributions across the intersected segment categories were done using: (a) crosstabs and their associated Cramer’s V tests for assessing relationships between climate change segment membership and COVID-19 vaccine membership (including within subgroups such as women participants); (b) Chi-squared tests for comparing cross-segment membership distributions between different genders, age groups, and political identities, where one category was used as the reference/expected distribution to assess differences between categories and values were standardized to account for differences in sample sizes across categories (e.g., different numbers in each age group); and (c) Chi-squared tests for analyzing the impact of interactions between gender and political identity, and between age and political identity, on cross-segment membership distributions (as per b). Post hoc power analyses were conducted using G*Power software (Version 3.1.9.6).

## Supplementary Information

Supplementary Information.

## Data Availability

The datasets used and/or analyzed during the current study are available from the corresponding author on reasonable request.
