# Supplementary material for: Comparative analysis of Australian climate change and COVID-19 vaccine audience segments shows climate skeptics can be vaccine enthusiasts
Source: Sci Rep. 2023 Jan 20;13:1118. doi: 10.1038/s41598-022-26959-5 (PMC9853496; doi:10.1038/s41598-022-26959-5)
Supplement: Supplementary file 1 — Supplementary Information. [file 41598_2022_26959_MOESM1_ESM.docx]

## Supplementary material

### Sample demographic characteristics

*Table S1*. Proportion of the sample within climate change segments with each socio-demographic characteristic.

|  | Alarmed | Concerned | Cautious | Disengaged | Doubtful | Dismissive |
| --- | --- | --- | --- | --- | --- | --- |
| *What is your gender?* | | | | | | |
| Men | 54% | 38% | 44% | 36% | 61% | 82% |
| Women | 45% | 60% | 56% | 64% | 39% | 18% |
| Other | 1% | 2% | 1% | 0% | 0% | 0% |
| *What is your age?* | |  |  |  |  |  |
| 18-19 years | 6% | 6% | 6% | 0% | 1% | 1% |
| 20-29 years | 13% | 23% | 21% | 25% | 14% | 2% |
| 30-39 years | 17% | 19% | 20% | 13% | 16% | 14% |
| 40-49 years | 17% | 17% | 15% | 18% | 12% | 21% |
| 50-59 years | 21% | 14% | 14% | 40% | 12% | 18% |
| 60-69 years | 16% | 11% | 8% | 4% | 22% | 23% |
| 70 years and above | 11% | 10% | 17% | 0% | 23% | 23% |
| *What is your highest completed education qualification?* | | | | | | |
| Did not go to school | 1% | 1% | 2% | 0% | 0% | 0% |
| Primary or high school | 31% | 51% | 47% | 61% | 48% | 45% |
| Trade/diploma/certificate | 33% | 22% | 26% | 34% | 34% | 34% |
| Bachelor degree or above | 34% | 26% | 25% | 5% | 18% | 21% |
| *Generally speaking, how would you describe yourself?* | | | | | | |
| Very conservative | 4% | 6% | 9% | 6% | 5% | 21% |
| Somewhat conservative | 21% | 23% | 25% | 5% | 27% | 16% |
| Moderate | 38% | 51% | 52% | 82% | 51% | 49% |
| Somewhat liberal | 27% | 16% | 10% | 4% | 15% | 4% |
| Very liberal | 10% | 5% | 3% | 4% | 2% | 10% |
| *Do you belong to a religion and, if yes, which religion do you belong to?*  (Aggregated from individual listing of 13 options) | | | | | | |
| No religion | 49% | 57% | 49% | 29% | 50% | 44% |
| Christian | 41% | 35% | 43% | 69% | 49% | 47% |
| Other | 10% | 8% | 7% | 2% | 1% | 6% |

*Table S2*. Proportion of the sample within COVID-19 vaccine segments with each socio-demographic characteristic.

|  | Enthusiast | Supporter | Social | Hesitant | Skeptic |
| --- | --- | --- | --- | --- | --- |
| What is your gender? | | | | | |
| Men | 71% | 53% | 36% | 27% | 30% |
| Women | 29% | 46% | 63% | 73% | 70% |
| Other | 1% | 2% | 1% | 0% | 0% |
| What is your age? | | | | | |
| 18-19 years | 3% | 4% | 9% | 8% | 3% |
| 20-29 years | 5% | 17% | 28% | 30% | 21% |
| 30-39 years | 6% | 18% | 24% | 25% | 30% |
| 40-49 years | 9% | 16% | 20% | 20% | 22% |
| 50-59 years | 21% | 17% | 13% | 12% | 13% |
| 60-69 years | 22% | 17% | 5% | 4% | 7% |
| 70 years and above | 34% | 12% | 3% | 1% | 4% |
| *What is your highest completed education qualification?* | | | | | |
| Did not go to school | 0% | 2% | 1% | 2% | 0% |
| Primary or high school | 34% | 39% | 53% | 60% | 55% |
| Trade/diploma/certificate | 29% | 28% | 27% | 25% | 31% |
| Bachelor degree or above | 37% | 32% | 19% | 13% | 14% |
| *Generally speaking, how would you describe yourself?* | | | | | |
| Very conservative | 5% | 5% | 14% | 5% | 11% |
| Somewhat conservative | 25% | 19% | 25% | 25% | 18% |
| Moderate | 36% | 49% | 47% | 65% | 61% |
| Somewhat liberal | 26% | 19% | 13% | 3% | 8% |
| Very liberal | 8% | 9% | 1% | 2% | 3% |
| *Do you belong to a religion and, if yes, which religion do you belong to?*  (Aggregated from individual listing of 13 options) | | | | | |
| No religion | 41% | 41% | 60% | 66% | 67% |
| Christian | 54% | 48% | 33% | 30% | 27% |
| Other | 5% | 12% | 7% | 5% | 6% |

### Climate change segmentation questions

How worried are you about climate change? (4-point Likert: Very worried to Not at all worried)

How much do you think climate change will harm you personally? (4-point Likert: Not at all to A great deal; plus Don't know)

How much do you think climate change will harm future generations of people? (4-point Likert; Not at all to A great deal; plus Don't know)

How important is the issue of climate change to you personally? (5-point Likert; Not at all important to Extremely important)

### COVID-19 segmentation questions

To what extent do you feel that getting a COVID-19 vaccine will be ...

- Bad-Good (7 points)
- Unpleasant-Pleasant (7 points)
- Harmful-Beneficial (7 points)
- Worthless-Valuable (7 points)
- Ineffective-Effective (7 points)
- Unsafe-Safe (7 points)
- Undesirable-Desirable (7 points)

When a coronavirus vaccine is available in Australia, what would you do?

- I will get vaccinated against the coronavirus Yes/No

When a coronavirus (COVID-19) vaccine becomes available: (Yes, definitely; Unsure, but leaning towards Yes; Unsure, but leaning towards No; No, definitely not)

- Would you accept the vaccine for yourself?
- Would you accept the vaccine if it meant protecting friends, family, or at-risk groups?
- Would you be willing to put your name on the list to be vaccinated first?

How much do you agree or disagree with the following statements: (5-point Likert: Strongly agree to Strongly disagree)

- Most of my family and friends will take a COVID-19 vaccine when available
- Most people who are important to me would approve of my getting a COVID-19 vaccine when available
- Doctors would think that I should get a COVID-19 vaccine when available
- I am confident that I will get a COVID-19 vaccine as soon as it is available in my area
- Whether I could get a COVID-19 vaccine is up to me.
